# Parents and Children During the COVID-19 Lockdown: The Influence of Parenting Distress and Parenting Self-Efficacy on Children’s Emotional Well-Being

**DOI:** 10.3389/fpsyg.2020.584645

**Published:** 2020-10-06

**Authors:** Mara Morelli, Elena Cattelino, Roberto Baiocco, Carmen Trumello, Alessandra Babore, Carla Candelori, Antonio Chirumbolo

**Affiliations:** ^1^Department of Dynamic and Clinical Psychology, Sapienza University of Rome, Rome, Italy; ^2^Department of Human and Social Sciences, University of Aosta Valley, Aosta, Italy; ^3^Department of Developmental and Social Psychology, Sapienza University of Rome, Rome, Italy; ^4^Department of Psychological, Health, and Territorial Sciences, D’Annunzio University of Chieti–Pescara, Chieti, Italy; ^5^Department of Psychology, Sapienza University of Rome, Rome, Italy

**Keywords:** pandemic, parents, children, psychological distress, parents’ self-efficacy, parenting, emotion regulation, COVID-19

## Abstract

On March 10, 2020, Italy went into lockdown due to the Coronavirus Disease-19 (COVID-19) pandemic. The World Health Organization highlighted how the lockdown had negative consequences on psychological well-being, especially for children. The present study aimed to investigate parental correlates of children’s emotion regulation during the COVID-19 lockdown. Within the Social Cognitive Theory framework, a path model in which parenting self-efficacy and parental regulatory emotional self-efficacy mediated the relationship between parents’ psychological distress and both children’s emotional regulation, and children’s lability/negativity, was investigated. A total of 277 parents of children aged from 6 to 13 years completed an online survey that assessed their psychological distress, regulatory emotional self-efficacy, and parenting self-efficacy. Parents reported also children’s emotional regulation and lability/negativity. A structural equation model (SEM) using MPLUS 8.3 was tested. Results showed that the hypothesized model exhibited excellent fit, chi-square (83) = 140.40, *p* < 0.01, RMSEA = 0.05, CFI = 0.97, TLI = 0.96, SRMR = 0.04. The influences of parents’ psychological distress and parents’ regulatory emotional self-efficacy on children’s emotional regulation and lability/negativity were mediated by parenting self-efficacy. The mediation model was invariant across children’s biological sex and age, and geographical residence area (high risk vs. low risk for COVID-19). Results suggested how parents’ beliefs to be competent in managing parental tasks might be a protective factor for their children’s emotional well-being. Implications for intervention programs are discussed.

## Introduction

The spread of the pandemic COVID-19 in Italy from February 2020 and the subsequent health emergency led to several restrictive measures. Schools and universities have been closed at the end of February, and starting from March 9th, 2020, a quarantine measure became necessary leading to a general closure of almost all public businesses and work activities.

Children and families have been deprived of their educational, work, and sport activities, but also from all their friendship and relational contacts. Suddenly parents had to manage their children at home from school 24 h a day and, at the same time, most of them had to start smart-working from home, still carrying out their children’s school commitments. Many parents also had to manage difficulties and pain related to having sick or dead relatives, having had wages reductions, or in some cases, having lost their work. It is easy to understand how Italian families have been exposed to a very strong emotional and psychological stress.

This situation had relevant repercussions on daily life of families, especially of children that have been deprived of their socialization and play spaces. The parents suddenly became the only point of reference for their children since the other references and educational figures were no longer available.

As enlightened by the World Health Organization (WHO, 2020a,b), this situation could have long-term negative consequences on psychological well-being and there is a need to invest in mental health services and other services. A recent review stressed how people all over the world can show many different psychological disorder symptoms related to the pandemic (Shahyad and Mohammadi, 2020). The WHO (2020a) highlighted that children were also showing signs of mental illness. In fact, both international and domestic studies showed that, during the lockdown, children exhibited several problems, such as anxiety and emotional and behavioral disorders (Jiao et al., 2020; Spinelli et al., 2020; Xie et al., 2020).

The European Pediatric Association–Union of National European Pediatric Societies and Associations (EPAUNEPSA; Jiao et al., 2020) has stressed the importance to address children’s psychological needs during the pandemic due to the negative repercussions on their psychological well-being, highlighting the relevant protective role of parents in decreasing their fear and stress. Research on psychological consequences of traumatic events, such as the terroristic attack of September 11, 2001, showed how children can also experience long-term effects on psychological well-being, reporting mental disorders after 6 months (Hoven et al., 2005).

All these data highlight the importance of not underestimating the psychological risks that children and their families could face. In a report on May 13th, 2020, the United Nations also underlined how, during the COVID-19 pandemic, the emotional problems of children and adolescents were exacerbated by family stress, social isolation, interruption of school and educational activity, and uncertainty for the future which occurred in critical moments of their emotional development (United Nations, 2020). Understanding how to strengthen parents and families in this situation, with the aim to protect children, represents an important goal that researchers should have in this period because it is possible that other future pandemics will affect humanity (Cluver et al., 2020).

The limited research conducted to date on the effects of the COVID-19 pandemic on well-being of parents and their children both in China and in Italy showed that COVID-19 related risks, such as (a) living in a red zone (i.e., a high-risk zone like Lombardia and Veneto for Italy), (b) being a parent positive to SARS-COV-2, (c) having relatives or friends positive to the SARS-COV-2 or who died from the SARS-COV-2, and (d) living in a high-risk environment (i.e., not having an open space in the home during the lockdown, losing a job during the pandemic, having a low income, not having internet connection), did not have strong negative direct effects on families’ well-being (Spinelli et al., 2020) or on children’s symptoms and problematic behaviors *per se* (Jiao et al., 2020). Actually, the research conducted by Spinelli et al. (2020) in Italy showed that it was the parenting stress related to the health emergency, the pandemic, and the lockdown that increased children’s psychological, emotional, and behavioral problems. In line with these findings, Wang et al. (2020) suggested the need to deeply understand the family functioning and processes that can promote children’s psychological well-being during the pandemic.

For this reason, this study focused on identifying which parental psychological variables can mediate the relationship between parents’ psychological distress during the pandemic and the lockdown and their children’s emotional regulation, in order to understand which possible intervention should be implemented to ameliorate families’ well-being. Two recent meta-analyses highlighted the relevant role that the parent–child relationship can have in promoting children’s effortful self-regulation (Pallini et al., 2018) and in decreasing children’s behavioral problems, specifically attention problems (Pallini et al., 2019).

The stress of quarantine can affect psychological well-being of adults, as confirmed in a recent review (Brooks et al., 2020), and might also have long-term effects (Liu et al., 2012). A study conducted on parents and children quarantined in 2009 during the H1N1 influenza showed that the high-stressful isolation increased parents’ psychological distress that in turn had an impact on their children’s well-being (Sprang and Silman, 2013). Children who have parents with high levels of stress showed more externalizing problems and developed less emotion regulation (Deater-Deckard and Panneton, 2017).

As reported by Leary and Hoyle (2009), psychological distress upsets the ability to self-regulate (Tillema et al., 2001; Scott and Cervone, 2002) but regulatory emotional self-efficacy is crucial in the self-regulation of relationships and behavior (Bandura et al., 2003). According to Bandura (1997), psychological distress, such as lack of social support or parental depression, can affect parenting self-efficacy, which is the belief that parents have to be able to manage their parental tasks successfully and that it is, in turn, related to children’s adjustment (Jones and Prinz, 2005).

Some previous studies showed that the relationship between parental mental health and children’s emotional and behavioral well-being is mediated by positive parenting strategies (Giallo et al., 2014). According to Eisenèberg et al. (2005), parents’ positivity and warmth can promote effortful control in children, reducing their externalizing behaviors. Likewise, self-efficacy, specifically parenting self-efficacy, can function as a mediator between environmental variables or psychological conditions related to an external situation (e.g., the stress related to the pandemic) and parenting competence. In fact, environmental aspects might also indirectly affect parents’ belief to be competent in managing parental tasks, and this could lead to less psychological well-being of the children (Jones and Prinz, 2005). For this reason, it is important that parents have a good parenting self-efficacy in order to display positive parenting strategies that can foster adaptive functioning and emotion regulation in children (Stack et al., 2010).

### Aims and Hypotheses

Within the theoretical framework of Social Cognitive Theory (Bandura, 1997), the present study aimed to investigate a path model in which parenting self-efficacy and parents’ regulatory emotional self-efficacy (related to COVID-19 lockdown) mediated the relationship between parents’ psychological distress and both children’s emotional regulation, and children’s lability/negativity, in line with a previous study that stressed how parenting self-efficacy can mediate the relationship between parents’ psychological distress and children’s adjustment (Giallo et al., 2014). Additionally, in our model parents’ psychological distress was also predicted by being exposed to several risks related to COVID-19 quarantine and the pandemic.

Moreover, the second aim was to assess whether children’s biological sex, children’s age, and geographical area (Northern Italy, which is the most at-risk area for the spread of the pandemic and for the risk of contagion, vs. the rest of Italy) moderated the structural paths of the model. There is evidence that children’s biological sex can affect parents’ way to respond to children (Sanders and Morawska, 2018) and that parenting self-efficacy can change over time (Deater-Deckard and Panneton, 2017), growing during early childhood (Weaver et al., 2008), and decreasing when children become adolescents (Glatz and Buchanan, 2015). Conversely, we did not expect to find any differences regarding living (or not living) in a high at-risk zone for the COVID-19 (i.e., Northern Italy), as found by recent Italian and Chinese studies (Jiao et al., 2020; Spinelli et al., 2020).

## Materials and Methods

### Participants

The present study was conducted in Italy, via an online survey, during April 2020 when there was a lockdown related to the health emergency due to the COVID-19 pandemic. Specifically, Italian parents with a child aged between 6 and 13 years were recruited through a snowball sampling procedure to complete the online survey. At the time of data collection, Italy had been in quarantine for more than 1 month. A link to the survey was shared among parents using different social networks (e.g., Facebook, WhatsApp), also asking parents to share the link among their contacts. Overall, 417 parents have had access to the survey, and a total of 277 valid questionnaires were used in the present investigation, yielding a response rate of 66.4%. Parents’ age ranged from 30 to 58 years old (M_*age*_ = 43.36, SD_*age*_ = 4.76) and the recruited sample mostly consisted of mothers (*n* = 248; 89.5%). As abovementioned, children were aged between 6 and 13 years (M_*age*_ = 9.66, SD_*age*_ = 2.29) and were almost equally distributed for biological sex (48% were boys and 52% were girls). Parents’ socioeconomic status (SES) was predominantly medium-high (92.1%; *n* = 255). 14.1% (*n* = 39) lived alone at home with children during the quarantine. 62% (*n* = 171) of parents were from northern Italy, the Italian area most affected by the pandemic, and 37.9% (*n* = 105) were from Central and Southern Italy which were areas less affected by the pandemic (although they were also put in lockdown). Regarding their work situation, 195 parents (70.4%) continued to work and earn as before the quarantine, while 82 parents (29.6%) lost their works or have had wage reductions or layoffs. 7.9% (*n* = 22) were health workers and 5.8% (*n* = 16) were health workers in a hospital department that treated SARS-COV-2-positive patients. 91.7% of parents (*n* = 254) did not have any relative tested positive for the SARS-COV-2, and 8.3% (*n* = 23) had at least one relative that tested positive for the SARS-COV-2. 96.8% (*n* = 268) have not-hospitalized relatives due to SARS-COV-2, and 3.2% (*n* = 9) have at least one hospitalized relative. Finally, 32.5% of parents (*n* = 90) did not have any acquaintance or a loved one that tested positive to SARS-COV-2, and 67.5% (*n* = 187) had at least one acquaintance or a loved one that tested positive for SARS-COV-2. Each parent gave his/her consent by clicking “Yes, I accept to participate in the study” on the first page of the survey. This study was approved by the Ethics Committee of Sapienza University of Rome, Department of Developmental and Social Psychology, protocol number: 427, April 16, 2020.

Two power analyses were conducted to determine the recommended minimum sample size: (1) for detecting a significant bivariate effect and (2) for conducting a structural equation model (SEM; Cohen, 1988). A moderate effect size of 0.25 was anticipated with a power level set at 0.80 and a significant alpha level set at 0.05. The minimum sample size necessary to detect a significant bivariate effect was *N* = 124. Regarding the SEM, with five latent and fifteen observed variables, using the software developed by Soper (2020), results indicated that the required minimum sample size to run a SEM and detect a significant effect was *N* = 229.

### Measures

#### COVID Risk Index

Using a similar procedure as used by Spinelli et al. (2020), we created an *ad hoc* index that assessed risks related to the COVID-19 pandemic. Specifically, a composite index was created given one point for each of the following risk factors, if present: (a) relatives that tested positive for SARS-COV-2, (b) friends or acquaintances that tested positive for SARS-COV-2, (c) hospitalized relatives due to SARS-COV-2, (d) living in northern Italy, which was the most at-risk area for the spread of the pandemic and for the risk of contagion, (e) being a health worker, and (f) being a health worker in hospital departments that treated SARS-COV-2 positive patients.

#### Family Risk Index

Again, using a similar procedure as used by Spinelli et al. (2020), we created an *ad hoc* index that assessed risks related to family situation during the quarantine and the pandemic. Specifically, a composite index was created given one point for each of the following risk factors if present: (a) a lower SES, (b) a worsened working situation during the quarantine, and (c) being a single or divorced parent who had to manage her/his own children at home alone during the quarantine. Both the family risk index and the COVID risk index are intended as summative rating scales that were created *ad hoc* for this research.

#### Parents’ Psychological Distress

Parents’ psychological distress during the lockdown was evaluated using the Perceived Stress Scale (Cohen et al., 1983; Italian validation by Mondo et al., 2019). Parents were asked to think about the last month. The scale is composed of 10 items that parents rated on 5 point-Likert scales from 1 (*never*) to 5 (*very often*). An example item is “During last month how do you usually feel nervous and stressed?” The scale showed a good reliability and validity also in the Italian validation (Mondo et al., 2019). In the present sample, the measure showed a good reliability, Cronbach’s alpha of 0.84.

#### Parents’ Regulatory Emotional Self-Efficacy

The Regulatory Emotional Self-Efficacy Scale (Caprara et al., 2013b) is a 13-item scale that evaluates the belief of parents to be able to manage with their negative emotions (i.e., anger, sadness, fear, and guilt) during the COVID-19 lockdown on a 5 point-Likert scale from 1 (*Not able*) to 5 (*Able*). The scale was modified asking parents to think about the quarantine period related to COVID-19 health emergency, and the following item was added to the scale “How do you feel able to manage the anxiety caused by hearing the news about coronavirus that is given on TV or that you read on the internet?” The scale showed good validity and reliability (Caprara et al., 2013a,b). In the present sample, the scale showed a good reliability: Cronbach’s alpha of 0.87.

#### Parenting Self-Efficacy

Parents completed the Parenting Self-Agency Measures (Dumka et al., 1996; Baiocco et al., 2017) which is an 8-item scale that evaluates the belief of parents to be able to manage with daily parental demands (i.e., feeling to be a good parent, working to face and solve difficulties with their children) during the month of lockdown on 7-point Likert scales from 1 (*seldom*) to 7 (*always*). The scale was modified, asking parents to think about the quarantine period related to COVID-19 health emergency, and three items were added to the original scale. These three items asked parents how they feel able to reassure their children about the health emergency, to organize their children’s daily life during the quarantine, and to explain to their children what is happening. The scale showed good validity and reliability (Baiocco et al., 2017, 2018). In the present sample, the scale showed a good reliability: Cronbach’s alpha of 0.87.

#### Children’s Emotion Regulation

Parents were asked to think about their child during the quarantine and to complete a short version of the Emotion Regulation Checklist (Molina et al., 2014). This is a 10-item scale that evaluates two sub-dimensions, namely, emotional regulation (i.e., positive emotions, being able to give voice to his/her negative emotions) and lability/negativity (i.e., anger, disruptive behaviors, excessive exuberance) of children during the COVID-19 lockdown on a 4 point-Likert scale from 1 (*Almost never*) to 4 (*Almost always*). The scale showed good validity and reliability (Molina et al., 2014; Di Maggio et al., 2016). In the present sample, both emotional regulation and lability/negativity scores showed acceptable reliability, respectively Cronbach’s alpha of 0.65 and 0.78.

### Data Analysis

Firstly, bivariate correlations among variables were calculated along with descriptives. Afterward, a mediation analysis with latent variables was performed via SEM, employing a parceling strategy (e.g., Bagozzi and Heatherton, 1994; Little et al., 2002). A parcel represents an aggregate of different items measuring a specific construct (Little et al., 2002; Coffman and MacCallum, 2005). Two or three parcels were constructed for each of the latent variables using the “item-to-construct” balance approach (Little et al., 2002), which means building each parcel by examining the item–construct relationships as represented by factor loadings in the item-level factor analyses (for a detailed description of this procedure, see Little et al., 2002). In such a way, parcels typically contained a balanced number of items and had comparable reliabilities. Therefore, our model comprised three latent variables with three parcels each and two latent variables with two parcels each. Summative indexes (such as the CRI and FRI) were treated as manifest variables.

Model fit was evaluated with the following indices: (a) the Comparative Fit Index (CFI); (b) the Tucker–Lewis index (TLI); (c) the root mean squared error of approximation (RMSEA); (d) and the standardized root mean square residual (SRMR). In general, for TLI and CFI, values between 0.90 and 0.95 are considered acceptable (e.g., Bollen, 1989; Byrne, 1994; Marsh et al., 2004) and values above 0.95 are deemed to be very good (Hu and Bentler, 1999). On the other hand, RMSEA and SRMR values smaller than (or equal to) 0.08 indicate a good fit (e.g., Bollen, 1989; Browne and Cudeck, 1993; Hu and Bentler, 1999; Marsh et al., 2004).

In order to evaluate the statistical significance of indirect effects, which represented the “mediated” effects, the bootstrapping procedure was used employing 5000 samples with replacement from the full sample to construct bias-corrected 95 percent confidence intervals (CI) (Preacher and Hayes, 2008; Hayes, 2009). Mediation typically occurs if the indirect effect is significant, that is, the zero value is not included in the CI (Preacher and Hayes, 2008; Hayes, 2009).

Finally, to test possible moderation effects of children’s biological sex and age, and living in a geographical area with high COVID-19 risk, a multigroup approach within SEM was employed as suggested by Baron and Kenny (1986). In this procedure, the invariance of the structural parameters of the proposed model was tested separately for (a) boys and girls; (b) different levels of children’s age; (c) geographical residential area, that is, living (vs. not living) in Northern Italy. A detailed description of the procedure will be given in the “Results” section (see also Sauer and Dick, 1993; Cattelino et al., 2019). All analyses were run with statistical software SPSS 25 and MPLUS 8.3.

## Results

### Correlations Among Variables

The family risk index was positively related with parents’ psychological distress and positively with lability/negativity. Parents’ psychological distress was negatively related with parenting self-efficacy, parents’ regulatory emotion self-efficacy, and children’s emotion regulation and was positively related with children’s lability/negativity. Parenting self-efficacy was positively related with parents’ regulatory emotion self-efficacy, and children’s emotion regulation, and was negatively related with children’s lability/negativity. Parents’ regulatory emotion self-efficacy was positively related with children’s emotion regulation and negatively related with children’s lability/negativity. Finally, children’s emotion regulation was negatively related with children’s lability/negativity. Correlations, means, and standard deviations are reported in Table 1.

**TABLE 1:** Correlations among variables.

	**1**	**2**	**3**	**4**	**5**	**6**	**7**	**8**	**9**	***M***	***SD***
1. Children’s age	1									9.66	2.29
2. Children’s biological sex	−0.02	1								–	–
3. Family risk index	0.10	−0.08	1							0.68	0.96
4. COVID-risk index	0.01	0.01	0.01	1						1.62	1.04
5. Parents’ psychological distress	−0.05	0.01	0.15**	0.11	1					2.83	0.59
6. Parenting self-efficacy	−0.07	0.01	−0.10	−0.04	−0.46**	1				5.37	0.89
7. Parents’ regulatory emotional self-efficacy	−0.02	0.01	−0.05	−0.11	−0.59**	0.48**	1			3.08	0.54
8. Children’s emotional regulation	−0.11	0.01	−0.08	−0.01	−0.27**	0.50**	0.20**	1		3.29	0.48
9. Children’s lability/negativity	−0.06	−0.08	0.14*	0.02	0.19**	−0.24**	−0.15*	−0.38**	1	1.75	0.58

*p < 0.05, **p < 0.01. Biological sex was coded as 0 = boys and 1 = girls.

### Mediation Model

A SEM was employed to test the hypothesized mediation model in which parenting self-efficacy and parents’ regulatory emotional self-efficacy (related to the COVID-19 lockdown) mediated the relationship between parents’ psychological distress and both children’s emotional regulation and children’s lability/negativity. Moreover, parenting self-efficacy mediated the relationship between parents’ regulatory emotional self-efficacy and both children’s emotional regulation and children’s lability/negativity.

In the present paper, the mediation analysis strategy recommended by James et al. (2006) was followed. In the first step, the mediation model was tested (i.e., model without the direct effects, indicated with M_*med*_). In the second step, a full model, including all the direct effects, was tested (indicated with M_*full*_). The two nested models were compared via the chi-square difference test, contrasting M_*med*_ with M_*full*_ (Δχ^2^, Satorra and Bentler, 2001). A non-significant Δχ^2^ would reveal that the full model does not significantly increase the fit and therefore the mediation model is to be preferred since it is more parsimonious.

The mediation model (M_*med*_) showed an overall good fit, chi-square (83) = 140.40, *p* < 0.01, RMSEA = 0.05, CFI = 0.97, TLI = 0.96, SRMR = 0.04. The full model including direct effects (M_*full*_) did not apparently improve the model fit, chi-square (79) = 134.52, *p* < 0.001, RMSEA = 0.05, CFI = 0.97, TLI = 0.96, SRMR = 0.04. In fact, the two models were contrasted, and the chi-square difference test was not significant, Δχ^2^ (4) = 5.88, *p* = 0.20. Therefore, the mediation model (M_*med*_) should be preferred due to being more parsimonious compared to the full model.

**FIGURE 1:**
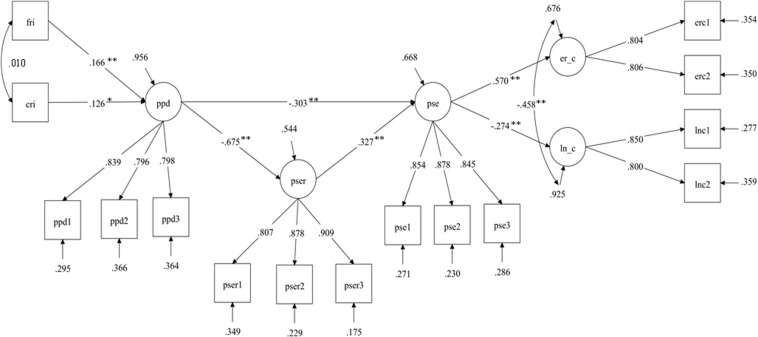
Multivariate mediation model with structural equation modeling. Standardized coefficients are reported. Fri, family risk index; Cri, COVID risk index; ppd, parents′ psychological distress; pser, parents′ regulatory emotional self-efficacy; pse, parenting self-efficacy; er_–_c, emotional regulation of children; ln_–_c, lability/negativity of children. All measurement parameters were statistically significant for *p <* 0.01. Fit Indexes Chi-square (83) = 140.40, *p* < 0.01, RMSEA = 05, CFI = 0.97, TLI = 0.96, SRMR = 0.04. *p < 0.05; **p < 0.01.

In Figure 1, all measurement and structural parameters of the mediated model (M_*med*_) are reported.

Parents’ psychological distress (ppd) was significantly, although modestly, affected by both COVID and family risk indexes. In turn, parents’ psychological distress significantly and negatively affected both parents’ regulatory emotional self-efficacy (pser) and parenting self-efficacy (pse). Parents’ regulatory emotional self-efficacy significantly and positively affected parenting self-efficacy. Finally, parenting self-efficacy positively and significantly influenced children’s emotional regulation (er_c) and negatively children’s lability/negativity (ln_c). More importantly, parenting self-efficacy mediated the effect of parents’ psychological distress and parents’ regulatory emotional self-efficacy on both children’s emotional regulation and children’s lability/negativity. With the exception of those involving the COVID and family risk indexes, all other indirect effects were statistically significant, supporting the mediated model (see Table 2). In Table 2, a full decomposition of total and specific indirect of the mediated model (M_*med*_) are reported. The COVID and family risk indexes displayed no significant effects on the other variables except for the aforementioned influence on parents’ psychological distress.

**TABLE 2:** Decomposition of indirect effects of the mediated model.

	**Effect**	**SE**	**Bootstrap 95% CI**
total indirect effect: ppd er_c	−0.30	0.05	[−0.39, −0.20]
specific indirect effect: ppd pse er_c	−0.17	0.06	[−0.29, −0.07]
specific indirect effect: ppd pser pse er_c	−0.13	0.04	[−0.20, −0.05]
total indirect effect: ppd ln_c	0.14	0.04	[0.06, 0.23]
specific indirect effect: ppd pse ln_c	0.08	0.03	[0.02, 0.16]
specific indirect effect: ppd pser pse ln_c	0.06	0.03	[0.02, 0.12]
indirect effect: pser pse er_c	0.19	0.06	[0.07, 0.29]
indirect effect: pser pse ln_c	−0.09	0.04	[−0.17, −0.02]

*All effects are standardized coefficients. If the zero value is not included in the bootstrap 95% CI, the effect is significant at *p* < 0.05. ppd, parents’ psychological distress; pser, parents’ regulatory emotional self-efficacy; pse, parenting self-efficacy; er_c, emotion regulation of children; ln_c, lability/negativity of children.*

Moreover, referring to the full model (M_*full*_), we also decomposed total, direct and indirect effects (see Table 3) with the aim to report the ratio of indirect to the direct effect and the proportion of mediated effect (MacKinnon et al., 1995).

**TABLE 3:** Decomposition of total, direct, and indirect effects in the full model.

	**Effect**	**SE**	**Bootstrap 95% CI**
ppd er_c			
total effect	−0.35	0.08	[−0.50, −0.17]
direct effect	−0.17	0.07	[−0.37, 0.02]
indirect effect	−0.18	0.10	[−0.32, −0.02]
ppd ln_c			
total effect	0.23	0.08	[0.07, 0.37]
direct effect	0.14	0.07	[−0.07, 0.37]
indirect effect	0.09	0.04	[0.009, 0.20]
pser er_c			
total effect	0.02	0.11	[−0.22, 0.23]
direct effect	−0.18	0.10	[−0.38, 0.01]
indirect effect	0.20	0.06	[0.07, 0.33]
pser ln_c			
total effect	−0.05	0.10	[−0.23, 0.15]
direct effect	0.02	0.10	[−0.17, 0.24]
indirect effect	−0.07	0.04	[−0.17, −0.003]

*All effects are standardized coefficients. If the zero value is not included in the bootstrap 95% CI, the effect is significant at *p* < 0.05. ppd, parents’ psychological distress; pser, parents’ regulatory emotional self-efficacy; er_c, emotion regulation of children; ln_c, lability/negativity of children.*

In regard to the relationship between ppd and er_c, the ratio of indirect to the direct effect was 1.06 (−0.18/−0.17 = 1.06), while with respect to the relationship between ppd and ln_c the ratio was 0.64 (0.09/0.14 = 0.64). In regard to the relationship between ppd and er_c, about the 51.42% of the effect was mediated (−0.18/−0.35 = 0.5142), while with respect to the relationship between ppd and ln_c about the 39.13% of the effect was mediated (0.09/0.23 = 0.3913).

In regard to the relationship between pser and er_c, the ratio of indirect to the direct effect was −1.11 (0.20/−0.18 = −1.11), while with respect to the relationship between pser and ln_c the ratio was −3.5 (−0.07/0.02 = −3.5). More problematic was to estimate, in the same fashion, the amount of mediated effect regarding the relationship between pser with both er_c and ln_c since in those cases the indirect and the direct effects have opposite signs (as can be seen in Table 3), and therefore, they tend to suppress each other, resulting in a reduced non-significant total effect (pser er_c:0.02 = −0.18 + 0.20; pser ln_c: −0.05 = −0.07 + 0.02). With respect to non-significant total effect, scholars have pointed out that mediation can occur also in the absence of a detectable total effect if the indirect effect is significant (e.g., MacKinnon, 2008; Hayes, 2009). This is apparently the case. In this perspective, although it cannot be absolutely claimed that effects were totally mediated and despite the presence of null total effects, it is worth to note that indirect effects were significant and that mediation has occurred.

Overall, we can conclude that the hypothesized mediation model (M_*med*_), reported in Figure 1, is consistent with the data. Moreover, the mediation did not fit significantly worse than the full model (M_*full*_) and therefore it was retained since it is more parsimonious (James et al., 2006). Additionally, all indirect effects of the mediated model (M_*med*_) were significant, indicating that mediation has occurred (e.g., Preacher and Hayes, 2008; Hayes, 2009).

### Multigroup Analysis

Within SEM, the test for a moderator effect can be performed using a multigroup analysis of the model in which the structural parameters are constrained equal across groups. Firstly, the structural parameters are freely estimated across groups to test for the baseline model. Secondly, the structural parameters are constrained to be equal across groups to test for the invariant model. In order to compare the fit of the two models, the chi-square difference test was used (Satorra and Bentler, 2001). A non-significant chi-square indicates that the parameters cannot be ruled out to be equal, then the invariant model should be retained and no moderation occurs. Instead, if the chi-square difference between the invariant and the baseline models is significant, which would mean that the invariant model fits significantly worse. Therefore, parameters are not equal across the groups and there is a moderation effect. Results of chi-square difference tests of multigroup analyses with SEM are reported in Table 4.

**TABLE 4:** Multigroup analyses for children’s biological sex and age.

	**χ^2^**	***p***	**χ^2^_*diff*_**
Children’s biological sex (Boys vs. Girls)			
Model 1: Baseline, Parameter Freely Estimated	χ^2^(134) = 185.88	<0.01	
Model 2: Invariant, Structural Parameter Constrained Equal	χ^2^(139) = 187.98	<0.01	
			χ^2^_*diff*_ (5) = 2.10; *p* = 0.83
Children’s age (6–10 y.o. vs. 11–13 y.o.)			
Model 1: Baseline, Parameter Freely Estimated	χ^2^(134) = 169.55	<0.01	
Model 2: Invariant, Structural Parameter Constrained Equal	χ^2^(139) = 179.32	<0.01	
			χ^2^_*diff*_ (5) = 9.77; *p* = 0.08
Geographical area (Northern Italy vs. Rest of Italy)			
Model 1: Baseline, Parameter Freely Estimated	χ^2^(134) = 172.56	<0.01	
Model 2: Invariant, Structural Parameter Constrained Equal	χ^2^(139) = 182.53	<0.01	
			χ^2^_*diff*_ (5) = 9.97; *p* = 0.08

Regarding children’s biological sex, the fit of the baseline model was chi-square (134) = 185.88, *p* < 0.01, RMSEA = 0.05, CFI = 0.97, TLI = 0.97, SRMR = 0.07, whereas the fit of the invariant model was chi-square (139) = 187.98, *p* < 0.01, RMSEA = 0.05, CFI = 0.98, TLI = 0.97, SRMR = 0.08. The chi-square difference test was not significant showing that the invariant model could not be rejected (Table 4). This finding suggests that biological sex of the children was not a moderator variable.

In regard to children’s age (6–10 y.o. vs. 11–13 y.o.), the fit of the baseline model was chi-square (134) = 169.55, *p* < 0.01, RMSEA = 0.04, CFI = 0.98, TLI = 0.98, SRMR = 0.06; conversely, the fit of the invariant model was chi-square (139) = 179.32, *p* < 0.01, RMSEA = 0.05, CFI = 0.98, TLI = 0.98, SRMR = 0.07. The chi-square difference test was not significant showing that the invariant model could not be rejected (Table 4). Therefore, children’s age did not appear to moderate the mediational effects tested in our model.

Finally, turning to geographical area (Northern Italy vs. rest of Italy), the fit of the baseline model was chi-square (134) = 172.56, *p* < 0.01, RMSEA = 0.05, CFI = 0.98, TLI = 0.98, SRMR = 0.06. Instead, the fit of the invariant model was chi-square (139) = 182.53, *p* < 0.01, RMSEA = 0.05, CFI = 0.98, TLI = 0.98, SRMR = 0.09. The chi-square difference test was not significant, showing that the invariant model did not fit significantly worse and therefore could not be rejected (Table 4). This result suggested that living in a high at-risk area for COVID-19, as it was Northern Italy at the time of data collection, did not significantly affect the structural parameter of our hypothesized model.

## Discussion

The health emergency related to the COVID-19 pandemic and the consequent restrictive measures of quarantine have upset our lifestyles and our daily life. In particular, families with children had to face an unprecedented and completely new situation in which parents suddenly remained the only reference point for their children. Results of the present study, indeed, showed that the COVID risk index and the family risk index partially contributed to the psychological distress of parents, although their impact was modest in terms of accounted variance. Specifically, parents with higher levels of psychological distress tendentially had a lower SES, had seen their working situation worsened during the quarantine, and were single or divorced parents who had to manage their children at home alone during the quarantine. Furthermore, regarding the COVID-19 risk index, parents with more psychological distress more likely had relatives, friends, or acquaintances tested positive for the SARS-COV-2, had hospitalized relatives because of the SARS-COV-2, lived in northern Italy which was the most at-risk area for the spread of the pandemic and for the risk of contagion, were health workers, and worked in hospital departments that treated SARS-COV-2-positive patients.

However, our SEM showed that parents’ psychological distress impacted on the emotional regulation and lability/negativity of their children passing through the mediators’ effect of parenting self-efficacy and parents’ regulatory emotion self-efficacy. These findings suggested that what could have a positive effect on children’s well-being and positive emotional regulation was not just being exposed to low level of parents’ psychological stress, but it was the fact that parents felt able to manage and carry out their parental role and the related tasks. Our results suggest that self-confident parents can successfully activate many personal resources that in turn seem to prevent their children’s emotional dysregulation, even in emergency situations such as the pandemic that increased their levels of psychological distress.

Furthermore, three multigroup analyses were performed to test the possible moderation effects of children’s biological sex and age and of geographical area (i.e., living or not living in Northern Italy, which is the most at-risk area for the spread of the pandemic and for the risk of contagion). The multigroup analyses showed that the hypothesized model was robust and invariant across children’s biological sex, and age, and living (or not living) in Northern Italy. Thus, in line with Spinelli et al. (2020), parents’ and children’s psychological distress was not affected by living in the high at-risk zone for COVID-19 (vs. not living in the high at-risk zone). We can speculate that, regardless of living in a more risky area, relationships among variables remained stable because this unprecedented situation characterized by the isolation and quarantine measures was perceived in the same way throughout Italy. Alternatively, it is also possible that our study did not have enough power to detect differences in parameters between groups.

Parents should be supported to improve their strengths and to feel able to manage their parental role and their emotions. During the quarantine, parents were the unique reference point for their children aged between 6 and 13 years who rely much on their parents in this life stage. It is important that parents know that they can protect their children, preventing their emotional dysregulation, using their strengths and self-confidence, even if they are experiencing fear and severe stress for the health emergency. Moreover, even if parents are exposed to high levels of stress, they can still promote a positive emotional functioning in their children if they feel able to reassure their children about the health emergency, to organize their children’s daily life during the quarantine, and to explain them what is happening.

Despite these important findings, this study had some limitations. We collected a convenience sample that was not representative of the Italian population. Moreover, emotion regulation and lability/negativity of children were reported by parents and this could be less informative. However, many other scholars have used this type of data collection which is very common in this kind of studies (e.g., Trumello et al., 2018; Spinelli et al., 2020). Moreover, our data are correlational and it is also conceivable that parental distress and self-efficacy could be affected by children’s lack of emotion regulation and lability/negativity. Furthermore, we assessed the parent’s own judgment of their children’s well-being and it is possible that parents who experience (according to themselves) a lot of distress also tend to judge their children’s well-being more negatively irrespective of the children’s actual well-being. Finally, it is not possible to infer causal relationships among variables because of the correlational nature of data. Future longitudinal study can be conducted in order to deeply test the possible long-term effects of parents’ psychological distress related to the health emergency on their children’s psychological well-being and the possible reverse causation effect.

However, despite these limitations, the present study presents many implications for prevention and intervention programs. In order to prevent children’s distress, intervention programs should start from family and parents. This programs should be aimed at increasing parents’ regulatory emotional self-efficacy and parenting self-efficacy, by activating their adaptive strategies and resources to deal with daily tasks and reinforcing their strengths. These parents’ skills could be taught and learned, representing an important resource even in emergency situations such as a pandemic, in which parents remain the only points of reference and education for their children. These prevention programs should be primarily addressed at (but not limited to) parents who are health workers, who lived alone with children during the quarantine, who have sick relatives, and who have a low SES and a worsened work situation, in order to prevent the impact of their psychological distress on their children, reinforcing their belief to be able to face this difficult situation and to manage both their parents tasks and their unavoidable negative emotions.

These findings suggest how clinicians should give psychological support to parents remotely during a lockdown, reinforcing their personal strengths and working on effective parenting and regulatory efficacy strategies. Indeed, parents with beliefs of self-efficacy in parenting behaviors and emotional regulation have children more emotionally regulated and psychologically healthy.

Likewise, the present results can be used to implement psychological and educational intervention for parents in order to prevent their children’s psychological distress. These results can also give pediatricians and psychologists important indications on how to specifically support families during the quarantine due to a global pandemic, providing advice to parents who in this period turn to pediatricians or psychologists to understand what to do to improve the well-being of their children. Telling parents that, even if they experience negative emotions, they can do a lot to help their children could empower parents, activating their skills and strategies. Intervention programs should be aimed to explain parents how to communicate to their children what is happening in the world around them. Using the correct words is more probable when parents have high levels of parental self-efficacy and emotional regulation self-efficacy (Jones and Prinz, 2005), and this could be very useful for parents’ and children’s well-being. Talking about the fear and the negative emotions related to the pandemic and the isolation would represent an important protective factor for families’ well-being. If parents understand which is the right way to communicate about the pandemic with their children, they can probably feel more self-confident in managing their parental tasks and their children’s emotion, and this aspect can have in turn positive effects on their children’s positive adjustment.

## Data Availability Statement

The raw data supporting the conclusions of this article will be made available by the authors, without undue reservation.

## Ethics Statement

The studies involving human participants were reviewed and approved by the Ethics Committee of the Department of Developmental and Social Psychology, Sapienza University of Rome. Written informed consent was not provided because data were collected via an online survey and participants were recruited via a snowball sampling. Thus, participants gave their informed consent by clicking “Yes, I accept to participate to this study” on the first page of the online survey.

## Author Contributions

MM, AC, EC, and RB conceptualized the study and organized the data collection. MM, AC, EC, RB, CT, AB, and CC collected the data. AC and MM run the analyses and wrote the methodological and results section. MM wrote the first draft of the manuscript. EC, CT, AB, and CC contributed to revision of the final version of the manuscript. All authors contributed to the article and approved the submitted version.

## Conflict of Interest

The authors declare that the research was conducted in the absence of any commercial or financial relationships that could be construed as a potential conflict of interest.
